# Prognostic Value of Sarcopenia in Patients With Diffuse Large B-Cell Lymphoma Treated With R-CHOP: A Systematic Review and Meta-Analysis

**DOI:** 10.3389/fnut.2022.816883

**Published:** 2022-02-25

**Authors:** Xin-Tian Xu, Dong-Liang He, Meng-Xing Tian, Hui-Jing Wu, Xin Jin

**Affiliations:** ^1^Department of Pharmacy, Hubei Cancer Hospital, Tongji Medical College, Huazhong University of Science and Technology, Wuhan, China; ^2^Department of Nutrition, Hengyang Central Hospital, Hengyang, China; ^3^Department of Clinical Nutrition, Hubei Cancer Hospital, Tongji Medical College, Huazhong University of Science and Technology, Wuhan, China; ^4^Department of Lymphoma Medicine (Breast Cancer and Soft Tissue Tumor Medicine), Hubei Cancer Hospital, Tongji Medical College, Huazhong University of Science and Technology, Wuhan, China

**Keywords:** sarcopenia, diffuse large B-cell lymphoma, overall survival, complete response, meta-analysis

## Abstract

**Objective:**

Several studies have reported conflicting results regarding the association between sarcopenia and outcomes in patients with diffuse large B-cell lymphoma (DLBCL). This meta-analysis aimed to evaluate the prognostic value of sarcopenia in patients with DLBCL.

**Methods:**

PubMed, Embase, and Cochrane Library databases were searched to identify trials exploring the association between sarcopenia and prognosis in patients with DLBCL treated with chemotherapy. A meta-analysis of overall survival (OS), progression-free survival (PFS), treatment completion, and rate of complete response (CR) was performed.

**Results:**

Twelve studies that involved 2,324 patients with DLBCL were included. Sarcopenia was associated with poor OS and PFS in patients with DLBCL, even after adjusting for confounders. Patients with sarcopenia had lower rates of CR and treatment completion than patients without sarcopenia.

**Conclusions:**

Sarcopenia is a negative predictor of prognosis in patients with DLBCL. Additional and prospective studies investigating the diagnostic criteria for sarcopenia are warranted.

## Introduction

Lymphomas are solid tumors in the immune system. Non-Hodgkin lymphoma accounts for ~90% of all lymphomas (1). Diffuse large B-cell lymphoma (DLBCL) is the most common type of non-Hodgkin lymphoma in the United States and worldwide (2, 3). Compared to the chemotherapy regimen of cyclophosphamide, vincristine, doxorubicin, and prednisone (CHOP), the combination of immunotherapy with rituximab (R-CHOP) has been found to significantly improve outcomes. Although progression-free survival (PFS) and overall survival (OS) have improved as validated by many randomized controlled trials, ~40% of patients experience relapse or progression (4). Clinicians and researchers have found that the prognosis of DLBCL is not only related to age, disease stage, and extranodal involvement but also closely to the patients' nutritional status and skeletal muscle loss.

Sarcopenia is defined as a progressive and generalized skeletal muscle disorder associated with an increased likelihood of adverse outcomes, including falls, fractures, physical disability, and mortality (5). Sarcopenia is prevalent in patients with cancer: 15–50% had skeletal muscle loss (6), while 38–70% were diagnosed with sarcopenia (6). Sarcopenia can increase the risk of death (7), reduce chemotherapy tolerance (8), increase the risk of postoperative complications, and reduce the quality of life (9) and survival (8, 10). Furthermore, several meta-analyses have also verified the prognostic role of sarcopenia in patients with lung cancer, ovarian cancer, gastric cancer, hepatocellular carcinoma, and head and neck cancer (11–15).

Recent studies have explored sarcopenia as a prognostic factor for patients with DLBCL. However, the results were inconsistent and controversial. One meta-analysis reported that sarcopenia predicted OS in patients with malignant hematological diseases, while only four studies on patients with DLBCL were included in the meta-analysis (16). Importantly, several recently published studies, which were not included in the above meta-analysis, further explored the prognostic role of sarcopenia in patients with DLBCL. To fill this knowledge gap, we conducted a comprehensive systematic review and meta-analysis of these studies. The impact of sarcopenia on clinical outcomes in patients with DLBCL undergoing immunochemotherapy was evaluated.

## Materials and Methods

### Search Strategy and Selection Criteria

This meta-analysis was conducted according to the Preferred Reporting Items for Systematic Reviews and Meta-Analyses statement. We searched all the published articles in the PubMed, Embase, and Cochrane Library databases until July 2021 for all references using the keywords, MeSH terms “sarcopenia” and “diffuse large B-cell lymphoma,” and other related words. The complete search used for PubMed was {[sarcopenia (MeSH Term)] OR muscle OR cachexia OR body composition} AND {[diffuse large B-cell lymphoma (MeSH Term)] OR [non-Hodgkin lymphoma (MeSH Term)]}. Unpublished studies and original data were not included. To avoid oversights in the literature search, two independent researchers searched for the relevant trials twice.

### Study Selection and Data Extraction

Studies were included if (1) the study was designed as a prospective cohort study or a retrospective study; (2) patients diagnosed with DLBCL were treated with chemotherapy; (3) skeletal muscle mass or function was measured before treatment; and (4) outcomes included OS, PFS, treatment completion, and rate of complete response (CR). Studies published as abstracts and case reports were excluded. Studies in which participants were not diagnosed with DLBCL and the diagnosis of sarcopenia was not clearly defined were also excluded.

Data from the included studies were extracted by two authors and checked by another author. The following data were collected: name of the first author, year of publication, characteristics of the study participants, number of participants, definition of sarcopenia, method to measure muscle, muscle measurement time, prevalence of sarcopenia, and anti-tumor therapy method for DLBCL and outcomes.

### Quality Assessment

The quality of the included trials was evaluated using the Quality In Prognostic Studies (QUIPS) tool by two reviewers independently—study participation, study attrition, prognostic factor measurement, outcome measurement, study confounding, and statistical analysis and reporting were assessed. If more than four of these six criteria had a low risk of bias, the study was considered to have a low risk of bias, and if two or more criteria had a high risk of bias, the study was considered to have a high risk of bias. The remaining studies were classified as having a moderate risk of bias (17).

### Data Synthesis and Statistical Analysis

Stata software (version 15.0, StataCorp., College Station, TX, USA) was used for statistical analyses. A heterogeneity test was performed for each analysis, and *I*^2^ > 50% indicated heterogeneity. When heterogeneity across studies was identified (*I*^2^ > 50%), the random-effects model was used to calculate the pooled hazard ratio (HR) and corresponding 95% confidence intervals (CIs). If studies were homogeneous, a fixed-effects model was used for the analysis. Most of the HR values were extracted from the univariate and multivariate Cox regression analyses, and a few were calculated using the Kaplan–Meier curves. If several methods were used to diagnose sarcopenia, such as skeletal muscle index (SMI) and skeletal muscle density (SMD), SMI was used in the meta-analysis for OS and PFS. A predefined subgroup analysis based on the anti-tumor treatment, skeletal muscle measurement method, and rate of sarcopenia was performed to identify the potential sources of heterogeneity and further explore the prognostic role of sarcopenia. Publication bias was assessed using. The funnel plots and Egger's regression intercept analysis.

## Results

### Study Selection and Characteristics

**Figure 1:**
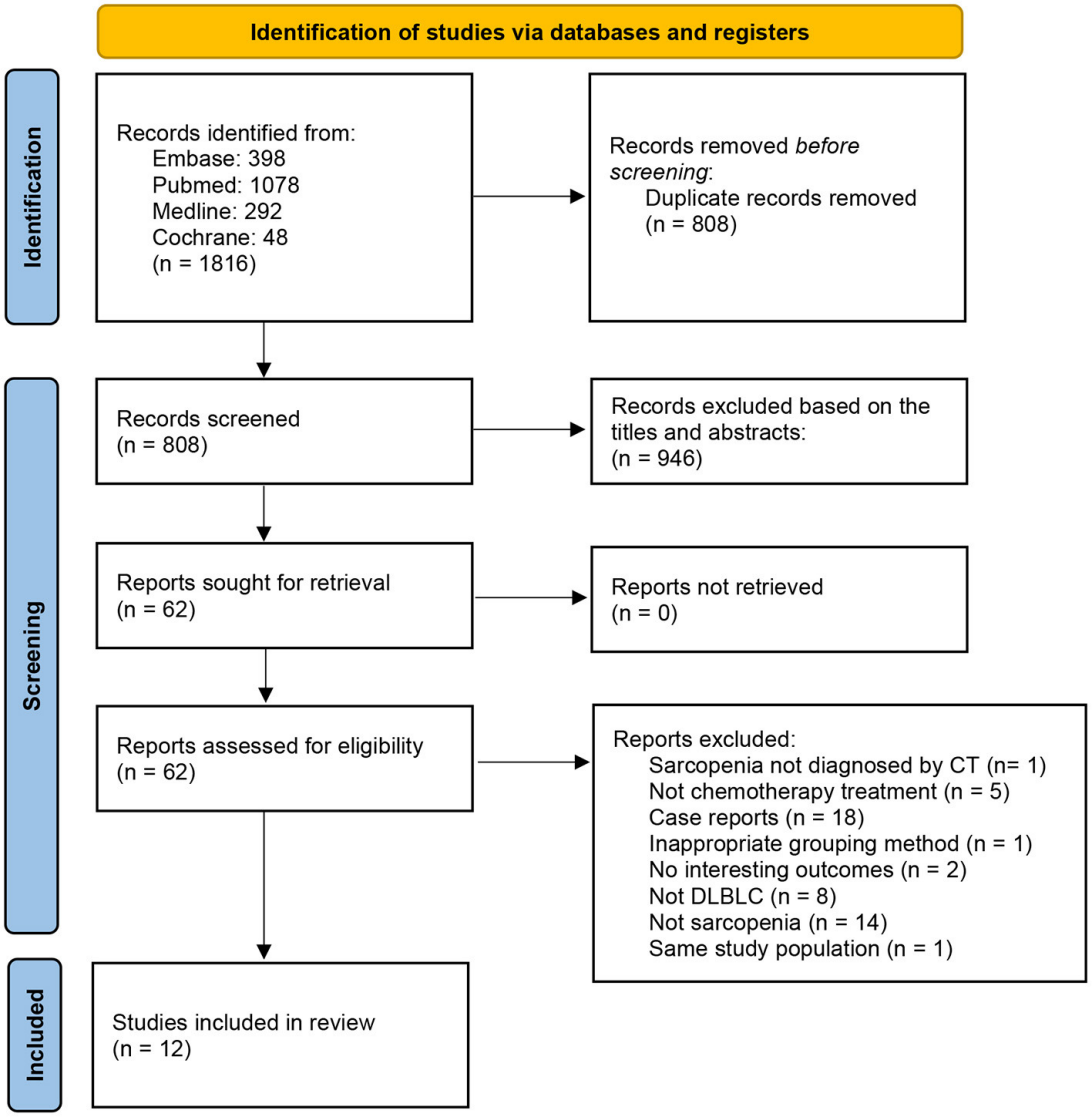
Flow diagram of the selection process in the meta-analysis.

A total of 659 related studies were extracted from the above-mentioned databases; 12 met the eligibility criteria and were thus included in the meta-analysis (Figure 1). The sample size ranged from 80 to 522 in the 12 studies. All included studies were retrospective cohort studies. The 12 included studies included 2,324 participants, among whom 996 were diagnosed with sarcopenia (18, 19). Seven studies used SMI at the third lumbar vertebra level (L3) on computed tomography (CT) (CT-L3-SMI) for the measurement of skeletal muscle mass (18–24). In the other three studies, SMD at the L3 level on CT imaging (CT-L3-SMD) was used for the measurement of skeletal muscle mass (18, 21, 25). In the two studies, skeletal muscle mass measured using SMI at the psoas level (CT-PM-SMI) (26) and fourth thoracic levels (CT-T4-SMI) (27) were used as the representative skeletal muscle mass of the whole body. One study used muscle mass at the L3 level on CT (CT-L3-muscle mass) as a diagnostic criterion for sarcopenia (28), while another study used a combination of CT-PM-SMI and CT-L3-SMI to diagnose sarcopenia (29). Eleven studies reported the OS and FPS. Five studies evaluated the impact of sarcopenia on treatment completion, and five studies assessed the rate of CR of anti-tumor therapy. The characteristics of the trials included in this meta-analysis are presented in Table 1.

**Table 1:** Characteristics of the trials included in the meta-analysis.

**References**	**Study design**	***n* (Male/female)**	**Age**	**Method to measure muscle**	**Definition of sarcopenia**	**Muscle measurement time**	**Prevalence of sarcopenia**	**Treatment**	**Outcomes**
Besutti et al. (18)	RS	60/56	63.7	CT-L3-SMI/CT-L3-SMD	L3-SMI: ♂ <43 cm^2^/m^2^ (BMI <25), ♂ <53 cm^2^/m^2^(BMI≥25), ♀ <41 cm^2^/m^2^, L3-SMD: <41 HU (BMI <25), <33 HU (BMI≥25)	Prior to treatment	25% (L3-SMI)	chemoimmunotherapy	OS, PFS (Univariate and multivariate analysis) Treatment completion
Camus et al. (19)	RS	35/45	78.66	CT-L3-SMI	♂ <55.8 cm^2^/m^2^, ♀ <38.9 cm^2^/m^2^	Prior to treatment	55%	R-CHOP/R-miniCHOP	OS FPS (Univariate and multivariate analysis) Treatment completion
Chu et al. (25)	RS	125/99	62	CT-L3-SMD	<41 HU (BMI <25), <33 HU (BMI≥25)	Within 1 months prior to treatment	51.8%	R-CHOP	OS FPS (Univariate and multivariate analysis) Treatment response
Go et al. (27)	RS	112/75	66.5/60	CT-T4-SMI	lowest quartile of T4 SMI	Prior to treatment	24.6%	R-CHOP	OS FPS (Univariate and multivariate analysis) Treatment completion Treatment response
Go et al. (20)	RS	112/81	NR	CT-L3-SMI	♂ <52.4 cm^2^/m^2^, ♀ <38.5 cm^2^/m^2^;	Prior to treatment	26.9%	R-CHOP	OS FPS Treatment response Treatment completion
Guo et al. (21)	RS	114/87	56.9	CT-L3-SMI/CT-L3-SMD	L3-SMI : <27.55 cm^2^/m^2^ L3-SMD: ≤ 36.86 HU	Within 4 months prior to treatment	23.9% (L3-SMI)	R-CHOP	OS FPS (Univariate and multivariate analysis)
Iltar et al. (26)	RS	66/54	59.11	CT-PM-SMI	♂ <440.4 mm^2^/m^2^ ♀ <306.87 mm^2^/m^2^	Prior to treatment	54.2%	R-CHOP	OS FPS (Univariate and multivariate analysis) Treatment response
Lanic et al. (22)	RS	36/46	78	CT-L3-SMI	♂ <55.8 cm^2^/m^2^, ♀ <38.9 cm^2^/m^2^;	Prior to treatment	54.9%	R-CHOP/R-miniCHOP	OS FPS (Univariate and multivariate analysis) Treatment completion
Nakamura et al. (23)	RS	121/86	60	CT-L3-SMI	♂ <47.1 cm^2^/m^2^, ♀ <34.4 cm^2^/m^2^	Prior to treatment	55.6%	R-CHOP/R-THP-COP	OS FPS (Univariate and multivariate analysis)
Rier et al. (28)	RS	80/84	64.5	CT-L3-muscle mass	Z-score < −1	Within 3 months prior to treatment	48.8%	R-CHOP	OS FPS (Univariate and multivariate analysis) Treatment response
Go et al. (29)	RS	130/98	64	CT-PM-SMI + CT-L3-SMI	L3-SMI: ♂ <52.4 cm^2^/m^2^, ♀ <38.5 cm^2^/m^2^, PM-SMI: ♂ <4.4 cm^2^/m^2^, ♀ <3.1 cm^2^/m^2^	Prior to treatment	43.9%	R-CHOP	OS FPS
Xiao et al. (24)	RS	510/12	68.1/61.2	CT-L3-SMI	♂ <53 cm^2^/m^2^, ♀ <41 cm^2^/m^2^	Within 3 months prior to treatment	49%	CHOP +/– R	Treatment completion

*♂:male; ♀: female; RS: retrospective study; R-CHOP: rituximab, cyclophosphamide, doxorubicin, vincristine, prednisone; R-THP-COP: rituximab, cyclophosphamide, tetrahydropyranyladriamycin, vincristine, prednisone*.

### Risk of Bias of Individual Studies

Table 2 presents an assessment of the risk of bias in the trials. According to the QUIPS checklist, five included studies had an overall low risk of bias, six trials had an overall moderate risk of bias, and one study had an overall high risk of bias.

**Table 2:** Quality assessment of individual studies using the QUIPS instrument.

**References**	**Study participation**	**Study attrition**	**Prognostic factor measurement**	**Outcome measurement**	**Study confounding**	**Statistical analysis and reporting**	**Overall risk of bias**
Besutti et al. (18)	M	L	L	L	L	M	M
Camus et al. (19)	M	L	L	L	L	M	M
Chu et al. (25)	L	M	L	L	L	L	L
Go et al. (27)	M	L	M	L	L	L	M
Go et al. (20)	M	L	L	L	H	L	M
Guo et al. (21)	M	L	L	L	L	M	M
Iltar et al. (26)	L	L	L	L	L	M	L
Lanic et al. (22)	L	L	L	L	L	M	L
Nakamura et al. (23)	L	L	L	L	L	M	L
Rier et al. (28)	M	L	L	L	L	M	M
Go et al. (29)	M	M	M	L	H	H	H
Xiao et al. (24)	M	M	L	L	L	L	L

*L, Low-risk; M, Moderate-risk; H, High-risk*.

### Association Between Pretreatment Sarcopenia and OS

**Figure 2:**
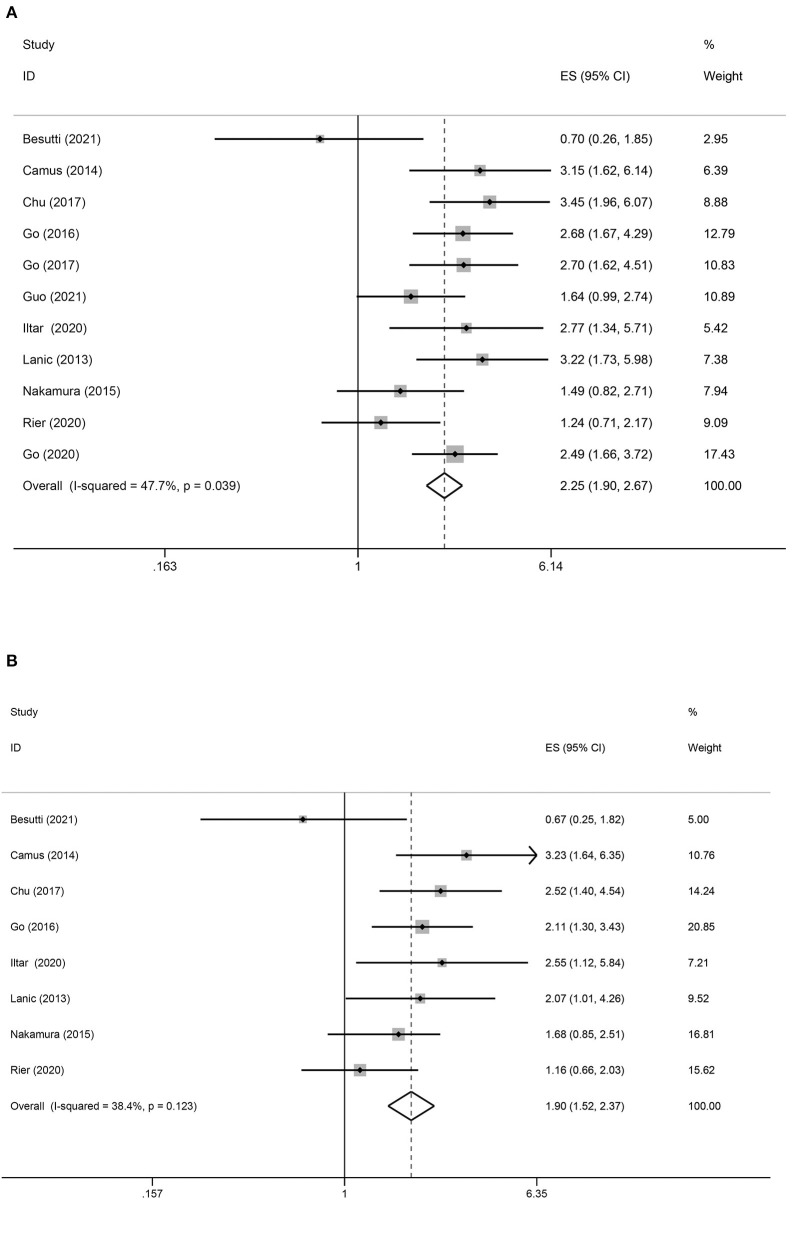
Forest plots of the association between sarcopenia and OS in patients with DLBCL undergoing chemotherapy. **(A)** Univariate analysis **(B)** Multivariate analysis. **(A)** Univariate analysis of pooled results of the association between sarcopenia and OS. The pooled HR was 2.25 (95% CI = 1.90–2.67, *p* < 0.01, *I*^2^ = 47.7%). **(B)** Multivariate analysis of pooled results of the association between sarcopenia and OS from. The pooled adjusted HR was 1.90 (95% CI = 1.52–2.37, *p* < 0.01; *I*^2^ = 38.4%).

In total, 11 studies reported OS as an outcome (Figure 2A). A fixed-effect model indicated moderate heterogeneity between studies (*I*^2^ = 47.7%), in which patients with sarcopenia tended to have a shorter OS than those without sarcopenia (HR = 2.25; 95% CI = 1.90–2.67, *P* < 0.01). According to the multivariate analysis of eight trials, the association between pretreatment sarcopenia and poor OS was significant (HR = 1.90; 95% CI = 1.52–2.37, *P* < 0.01; *I*^2^ = 38.4%) (Figure 2B).

To comprehensively evaluate the association between sarcopenia and OS in DLBCL, subgroup analysis was performed based on the anti-tumor treatment, skeletal muscle measurement, and rate of sarcopenia. In most subgroup meta-analyses, the pooled data indicated an association between sarcopenia and shorter OS in patients with DLBCL. Subgroup analysis also showed that sarcopenia had no significant impact on OS of patients with DLBCL treated with multiple treatment methods (HR = 1.21; 95% CI = 0.73–2.02, *P* > 0.05; *I*^2^ = 39.8%; Table 3).

**Table 3:** Subgroup analysis of the association between sarcopenia and survival of patients with DLBCL treated with chemotherapy.

**Subgroup**	**Methods**	**Heterogeneity**	**HR**	**95% CI**	***p***
**OS**					
**Anti-tumor treatments**					
R-CHOP	Fixed	28.2%	2.43	(2.03–2.91)	*p* < 0.001
R-CHOP + others	Fixed	39.8%	1.21	(0.73–2.02)	*p* = 0.456
**Measurement of skeletal muscle**					
CT-L3-SMI	Random	55.1%	2.03	(1.39–2.97)	*p* = 0.001
CT-L3-SMD	Random	0%	3.51	(2.47–4.98)	*p* = 0.029
Other measurement methods	Random	45.1%	2.20	(1.55–3.13)	*P* < 0.001
**Rate of sarcopenia**					
<30%	Random	60.8%	1.93	(1.22–3.06)	*p* = 0.005
>40%	Random	45.7%	2.36	(1.76–3.18)	*p* < 0.001
**PFS**					
**Anti-tumor treatments**					
R-CHOP	Fixed	0%	2.17	(1.85–2.56)	*p* < 0.001
R-CHOP + others	Fixed	0%	1.25	(0.85–1.85)	*p* = 0.250
**Measurement of skeletal muscle**					
CT-L3-SMI	Random	43.8%	1.95	(1.58–2.41)	*p* < 0.001
CT-L3-SMD	Random	0%	2.48	(1.84–3.36)	*p* = 0.001
Other measurement methods	Random	31.6%	2.01	(1.59–2.54)	*p* < 0.001
**Rate of sarcopenia**					
<30%	Fixed	0.5%	2.19	(1.72–2.78)	*p* < 0.001
>40%	Fixed	38.2%	1.89	(1.56–2.29)	*p* < 0.001

### Association Between Pretreatment Sarcopenia and PFS

**Figure 3:**
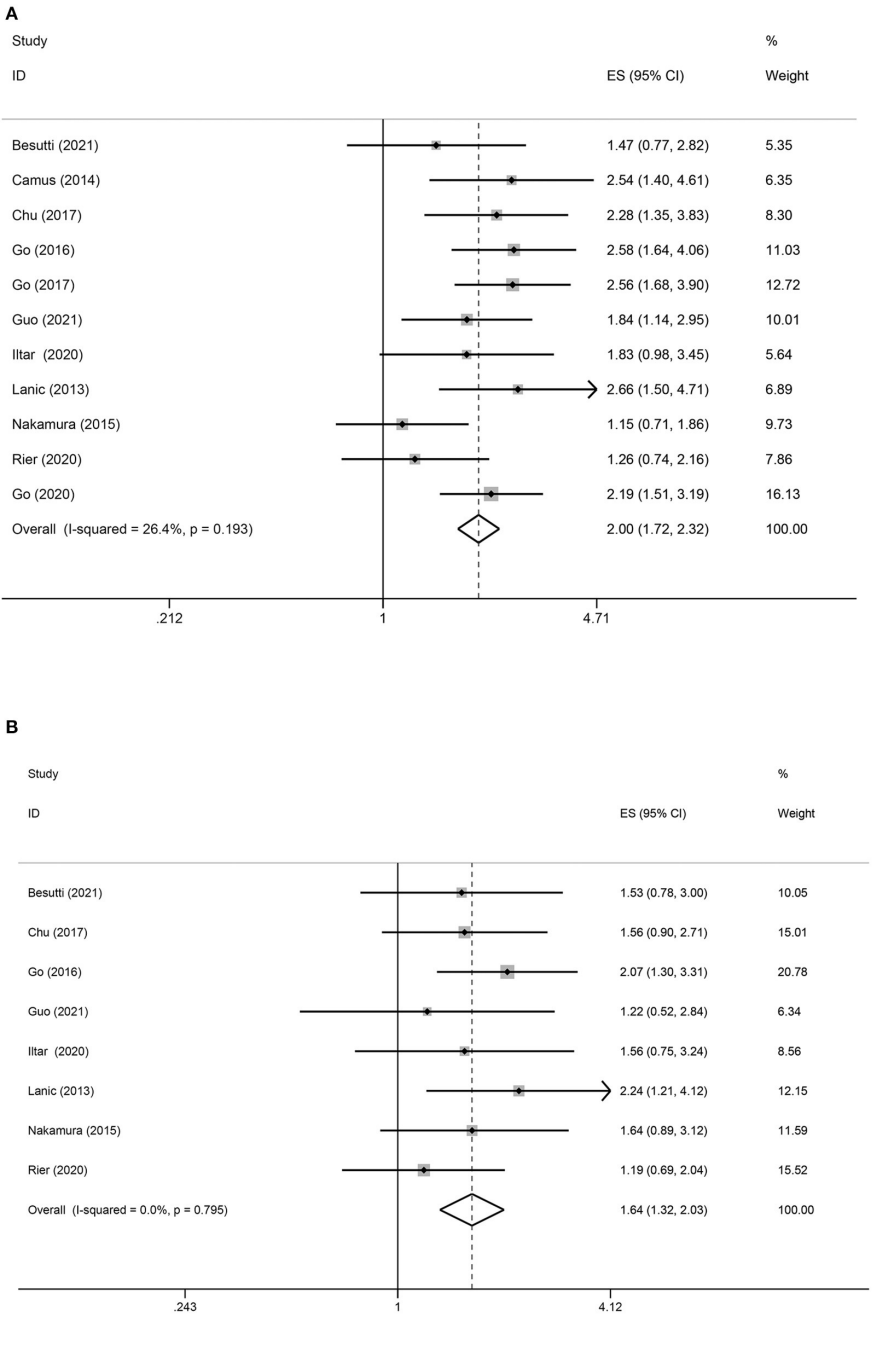
Forest plots of the association between sarcopenia and PFS in patients with DLBCL undergoing chemotherapy. **(A)** Univariate analysis **(B)** Multivariate analysis. **(A)** Univariate analysis of pooled results of the association between sarcopenia and PFS. The pooled HR was 2.00 (95% CI = 1.72–2.32, *p* < 0.01, *I*^2^ = 26.4%). **(B)** Multivariate analysis of pooled results of the association between sarcopenia and OS. The pooled adjusted HR in total was 1.64 (95% CI = 1.32–2.03, *p* < 0.01; *I*^2^ = 0.0%).

Eleven studies reported an association between pretreatment sarcopenia and PFS in patients with DLBCL. The crude pooled HR of skeletal muscle mass loss for PFS was 2.00 (95% CI = 1.72–2.32, *P* < 0.01), while low, non-significant heterogeneity was detected (*I*^2^ = 26.4%; *P* = 0.08). The adjusted summary HR from eight selected trials was 1.64 (95% CI = 1.32–2.03, *P* < 0.01), in which low, non-significant heterogeneity was detected (*I*^2^ = 0.0%) (Figure 3).

Subgroups analysis based on the anti-tumor treatments suggested that sarcopenia predicted negative PFS in patients with DLBCL treated with R-CHOP (HR = 2.17; 95% CI = 1.85–2.56, *P* < 0.01; *I*^2^ = 0%) but not in those treated with multiple treatment methods (HR = 1.25; 95% CI = 0.85–1.85, *P* = 0.250; *I*^2^ = 0%). The other subgroup analyses showed an association between sarcopenia and poor PFS (Table 3).

### Sarcopenia and Treatment Completion

**Figure 4:**
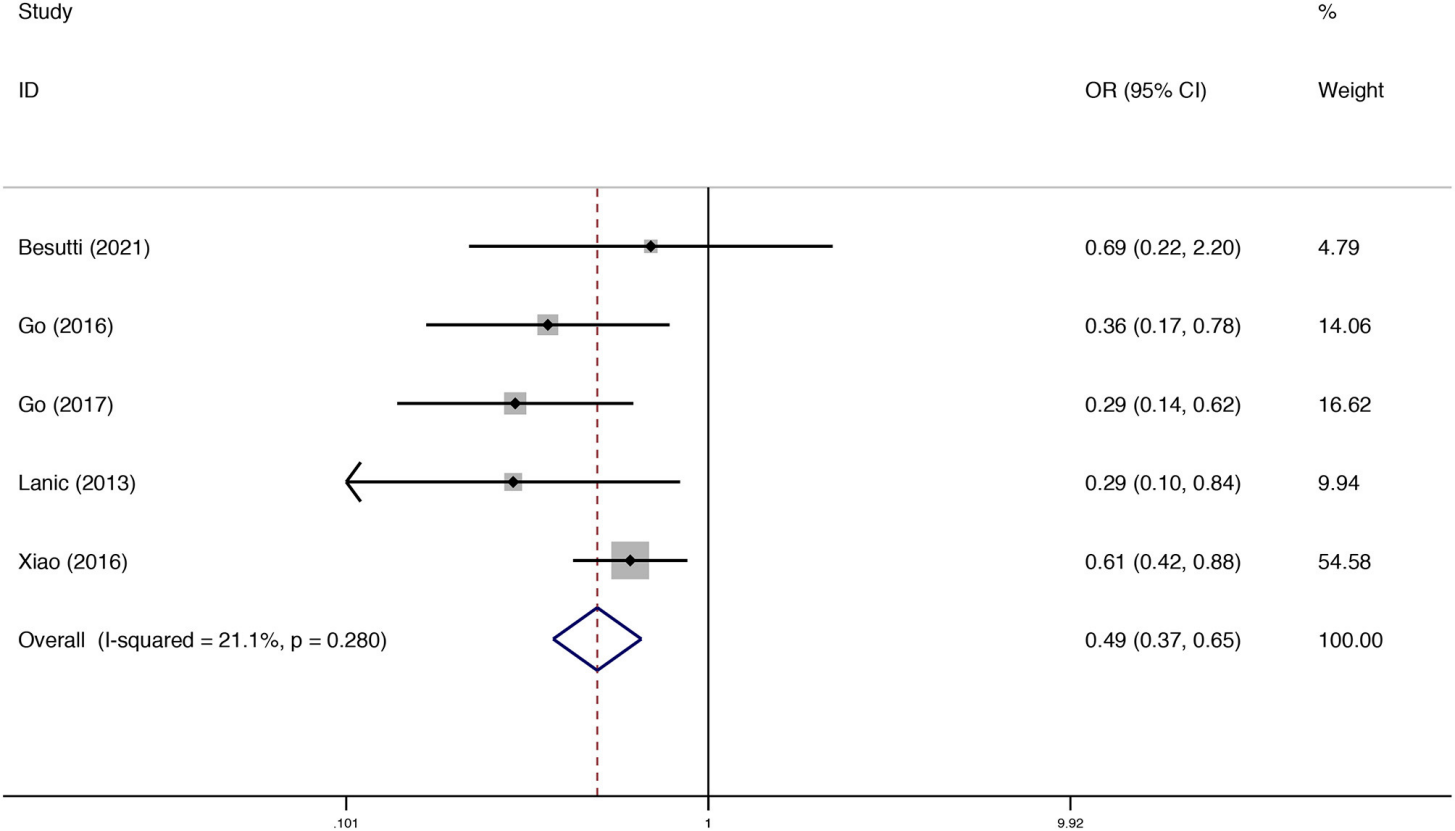
Forest plots of the meta-analysis of the effect of sarcopenia on treatment completion in patients with DLBCL undergoing chemotherapy. Pooled results of the OR of the rate of treatment completion in patients with DLBCL. The pooled OR of patients with DLBCL was 0.49 (95% CI = 0.37–0.65, *I*^2^ = 21.1%).

Five studies assessed the association between sarcopenia and treatment completion. The pooled results from the fixed model indicated that sarcopenia decreased the rate of treatment completion [odds ratio (OR) = 0.50; 95% CI = 0.37–0.65, *P* < 0.01]. Heterogeneity between studies was low (*I*^2^ = 21.1%) (Figure 4).

### Sarcopenia and Rate of CR

**Figure 5:**
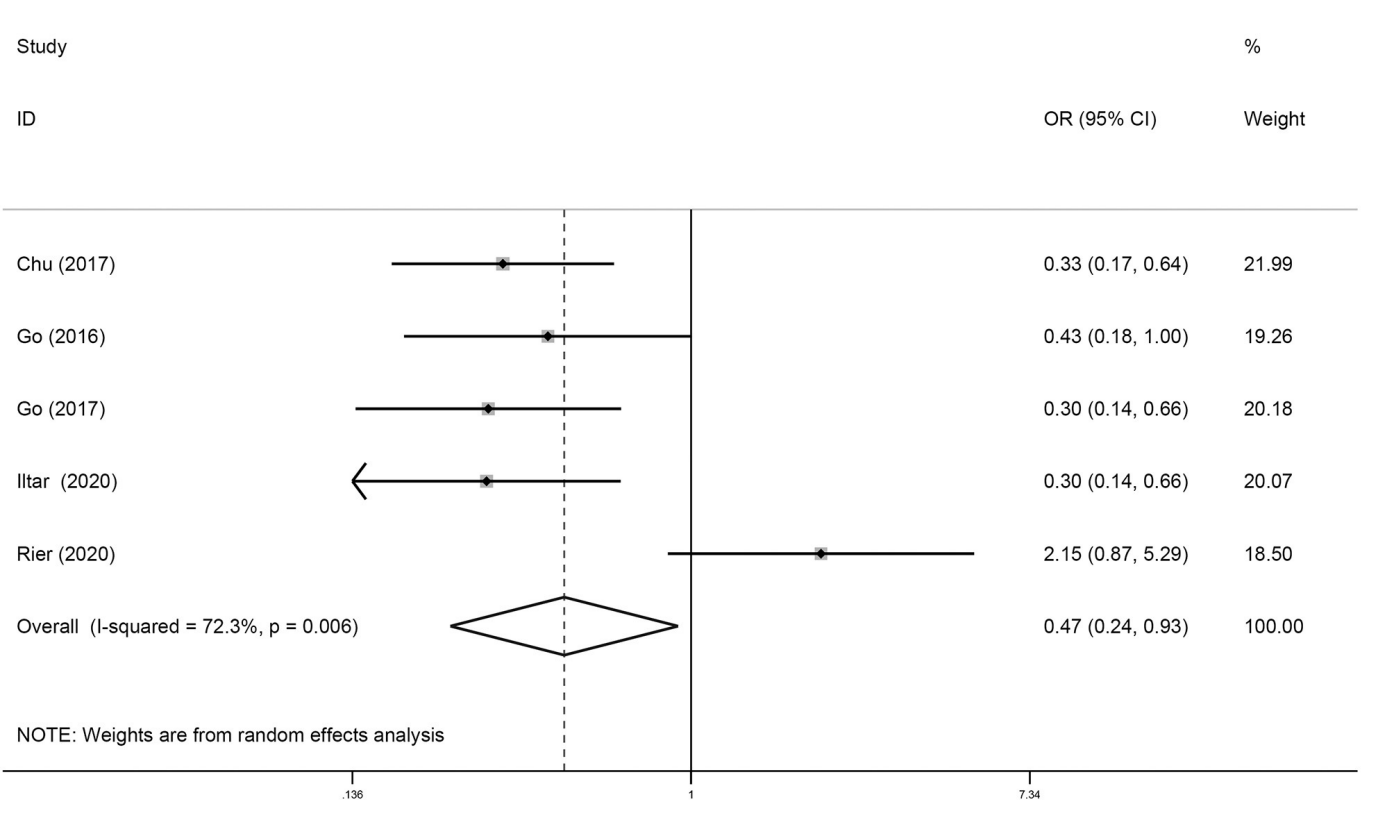
Forest plots of the meta-analysis of the effect of sarcopenia on the rate of CR in patients with DLBCL undergoing chemotherapy. Pooled results of OR of rate of CR in patients with DLBCL. The pooled OR of patients with DLBCL was 0.47 (95% CI = 0.24–0.93, *I*^2^ = 72.3%).

Five studies analyzed the relationship between sarcopenia and the rate of CR in DLBCL. As shown in Figure 5, sarcopenia predicted a low rate of CR (OR = 0.47; 95% CI = 0.24–0.93, *P* < 0.01). Howev er, there was significant heterogeneity between studies (*I*^2^ = 72.3%).

### Publication Bias

**Figure 6:**
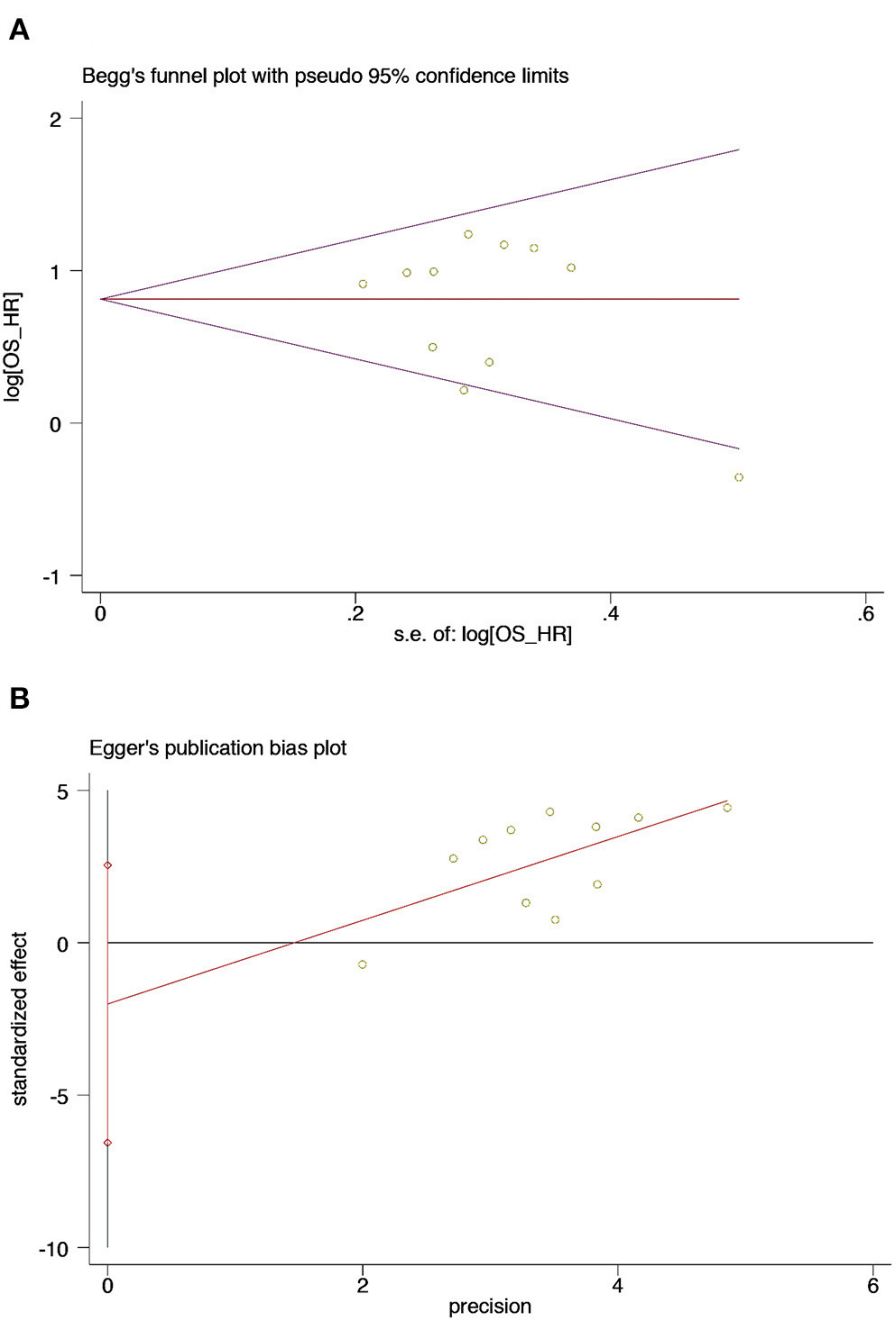
Begg's test and Egger's test of sarcopenia and OS from univariate analysis in patients with DLBCL undergoing chemotherapy **(A)** Begg's test **(B)** Egger's test. **(A)** Begg's funnel plots of publication bias for the association between sarcopenia and OS from univariate analysis (*p* = 0.755). **(B)** Egger's test of publication bias for the association between sarcopenia and OS from univariate analysis (*p* = 0.344).

The Begg's funnel plots and Egger's publication bias plots were used to assess the potential publication bias for OS in the univariate analysis. No publication bias was detected using the Egger's test (*P* = 0.344) (Figure 6).

## Discussion

Our meta-analysis showed that sarcopenia was associated with poor survival, even after adjustment for confounders. Furthermore, the meta-analysis outcomes showed a low rate of CR in patients with sarcopenia after R-CHOP therapy. In addition, patients with sarcopenia tended to fail to complete the treatment plan compared to patients without sarcopenia. These results are consistent with a recently published meta-analysis study, which demonstrated that overall mortality of hematopoietic cancer was significantly associated with sarcopenia. However, only two studies that involved patients with DLBCL were included, and several recently updated researches were not (30).

In our study, the incidence of sarcopenia ranged from 23.9 to 55.6%, which was consistent with the previous study involving United States veterans with DLBCL, which was >30% based on CT diagnosis before chemotherapy (24). The significant differences in the incidence of sarcopenia among patients with DLBCL were due to the lack of uniform diagnostic criteria and cut-off values. Additionally, patients with DLBCL also experience further muscle loss during chemotherapy, with skeletal muscle area decreasing by ~2.8% after treatment (24).

The mechanism of sarcopenia is complex. Aging is a crucial risk factor for sarcopenia. The aging process breaks the balance between muscle protein synthesis and catabolism, eventually leading to gradual loss of skeletal muscle loss (31). The mechanism of aging related sarcopenia also involves negative protein turnover characterized by reduction of myofibrillar and mitochondrial protein synthesis and increased proteolysis via the ubiquitin proteasome and calcium-dependent activation of proteases (32, 33). The decreased number of type II fiber satellite cells and the intramuscular and intermuscular fat infiltration (myosteatosis) caused by aging contribute to sarcopenia at the cellular level (31, 34). Systemic inflammation is also crucial for the pathogenesis of muscle loss in later life. The chronic pro-inflammatory state caused by increased production of pro-inflammatory cytokines is a possible underlying cause of muscle loss (35). The high incidence of sarcopenia in patients with DLBCL can be explained by the average age of the study cohort included in this meta-analysis, which ranged from 60 to 70 years. These patients are susceptible to sarcopenia. Moreover, various pro-inflammatory cytokines, including prostaglandins, interleukin-6, interleukin-1, tumor necrosis factor, interferon gamma, and leukemia inhibitory factor, secreted by the tumors also elicit catabolism in the skeletal muscles and accelerate muscle loss (36, 37). In addition, the abnormal metabolism of proteins and amino acids caused by the tumor exacerbates muscle loss. Malnutrition due to the tumor and anti-tumor therapy also affects muscle mass in patients with DLBCL (38). Furthermore, physical activity is also greatly limited in patients with cancer.

Our findings suggested an overall negative effect of sarcopenia in the rate of CR. However, this result was inconsistent with the previous studies. In the study by Rier, when low muscle mass (LMM) was used as the diagnostic criteria for sarcopenia, there was no difference in the rate of CR between the sarcopenia and non-sarcopenia groups (28). Another study showed that there was no difference in the rate of CR between the groups in patients whose chemotherapy treatment was uninterrupted (29). Upon reviewing the literature, we found the decreased clearance of the anti-tumor drugs in the tissues of patients with muscle reduction may improve the therapeutic effect while accompanying side effects, including increased toxicity (39). The study by Guo confirmed that grade 3–4 toxicity during or after immunochemotherapy was associated with poor body composition. For every 5 cm^2^/m^2^ decrease in SMI, the risk of any grade 3–4 toxicity increased by 34% (21). Another study also confirmed that the sarcopenia group had more frequent grade 3 anemia, grade 3–4 and 4 thrombocytopenia, and grade 4–5 non-hematologic toxicity (21). Increased toxicity may lead to early discontinuation of treatment, which may be a reason for the reduction in the rate of CR. The pooled data from this meta-analysis also indicated that sarcopenia was associated with the inability to complete the standard number of treatment cycles due to toxicity. Moreover, the association between lower SMG, SMD, SMI, and lean body mass (LBM) and the increasing risk of dose delay/reduction was also demonstrated in the study by Guo (21).

Most importantly, the meta-analysis provided convincing evidence that sarcopenia was associated with low OS and PFS. The association between sarcopenia and prognosis was independent of other prognostic factors, such as age, sex, prognostic index, and hypoalbuminemia. The pathogenesis of poor survival in patients with cancer and sarcopenia is unclear. A commonly postulated mechanism is that greater drug toxicity results in decreased treatment tolerance and reduction in effective drug doses (15, 40). Another potential explanation is that decreased levels of insulin-like growth factor-1 (IGF-1) are secreted by the smaller skeletal muscle and that both insulin-like growth factor-1 receptor (IGF-1R) density and signaling are impaired in the aged skeletal muscle (41). Recent studies have suggested that the IGF-1/IGF-1R signaling pathway may contribute to the progression of DLBCL and other cancers (42–44). In addition, muscle loss is a manifestation of malnutrition. Malnutrition encountered in patients with DLBCL also leads to increased incidence of treatment-related toxicity, which influences the occurrence of poor survival outcomes (38). Sarcopenia is also a hallmark of cancer cachexia. Poor OS and PFS may be a consequence of cancer cachexia rather than sarcopenia. Patients with cancer cachexia tend to have progressive functional impairment and worse clinical outcomes due to the profound systemic inflammation associated with cancer.

Although sarcopenia was associated with poor survival in patients with DLBCL, the prognostic value of low body mass index (BMI) in the included studies remains controversial. Two studies showed that low BMI was associated with poor OS and PFS (19, 22). However, in the four studies, underweight was not significantly associated with poor survival (20, 26, 27, 29). In Besutti's study, no significant difference was observed between different the BMI groups (<25, 25–30, ≥30) in terms of OS and PFS. However, sarcopenia in patients with obesity have the worst survival after further stratifying patients into sarcopenia and obesity group (18). As a result, the prognostic value of sarcopenia in DLBCL cancer patients with obesity, normal weight, and underweight should be further studied.

Our study has some limitations. All included trials were retrospective studies. There was considerable heterogeneity in the meta-analysis with respect to patient cohorts as a result of using different diagnostic criteria for sarcopenia. Although the muscle mass in all included studies was measured on CT, the scan level of CT used in each study was different, and the cut-off values varied. In addition, the various treatment strategies used in the studies were also likely to contribute to heterogeneity. Another limitation of our meta-analysis is the different definitions of OS and PFS in the studies. Further, the lack of measurement of relevant indicators of muscle function is a limitation. The included studies were retrospective and evaluated muscle mass only on CT, but not by muscle function. The definition of sarcopenia includes not only the reduction of muscle mass but also the degeneration of muscle function. Moreover, gene mutations also play a key role in determining the prognosis of patients with DLBCL. However, none of the included articles investigated the effect of sarcopenia on prognosis in patients with DLBCL with different gene mutations. Therefore, further studies using rigorous design are warranted to verify the strength of prognostic role of sarcopenia in patients with DLBCL.

## Conclusion

The prevalence of sarcopenia is higher in patients with DLBCL than in the general population. Loss of skeletal mass is associated with poor survival in patients with DLBCL. Sarcopenia also negatively affects the rate of treatment completion and CR to immunochemotherapy. Identification of consensus diagnostic criteria for sarcopenia and design of prospective studies that incorporate measurements of muscle strength and physical function are the areas for further research.

## Data Availability Statement

The original contributions presented in the study are included in the article/supplementary material, further inquiries can be directed to the corresponding author.

## Author Contributions

XJ: conceptualization, methodology, software, investigation, and writing—original draft. X-TX: methodology, software, and investigation. M-XT: resources, data curation, and investigation. D-LH and H-JW: writing—review and editing. All authors revised and approved the final manuscript.

## Funding

This study was supported by the Provincial Natural Science Foundation of Hunan (2021JJ30066).

## Conflict of Interest

The authors declare that the research was conducted in the absence of any commercial or financial relationships that could be construed as a potential conflict of interest.

## Publisher's Note

All claims expressed in this article are solely those of the authors and do not necessarily represent those of their affiliated organizations, or those of the publisher, the editors and the reviewers. Any product that may be evaluated in this article, or claim that may be made by its manufacturer, is not guaranteed or endorsed by the publisher.
